# Inhalation of Ethereal Vapor for the Purpose of Inducing Sensibility to the Pain of Surgical Operations

**Published:** 1846-12

**Authors:** 


### Miscellaneous Notices.

*Inhalation of Ethereal Vapor for the purpose of inducing insensibility to the pain of Surgical Operations.*—Great interest has been excited, both in professional circles and in the public mind, by an announcement in the Boston Medical and Surgical Journal, that a Mr. Morton of Boston had discovered a gaseous preparation of a nature so exquisitely anodyne as to fulfil the great desideratum in surgery; being capable of rendering a patient temporarily insensible to the severest pain. What every body heartily wishes to be true, is easily believed, and we do not wonder that a joyful expectation of future exemption from suffering has been widely indulged, and Mr. Morton's assumed discovery hailed with gratitude and felicitation. Such has been the case. Surgeons of eminence have attested the virtues of the article; physicians of distinction have trumpeted its praises; the patent office has secured the prospective emoluments of the sale to the fortunate inventor; and the industrious agents, hurried by the mighty power of steam, are already dispensing the ethereal vapor to all who have faith enough to breathe, and money enough to pay for it.

The most extraordinary quality of the article, which, by the by, is nothing more nor less than the vapor of sulphuric ether, is that it is capable of making those who handle it forget things apparently incapable of obliteration from memory, while it keeps the mind continually active in other respects. For instance, by being present at its administration several times, the surgeons of Mass. General Hospital, have apparently undergone a loss of memory, remarkable and painful. They seem to have forgotten the well known properties of ethereal vapor, as previously developed, together with all those long established rules of professional conduct which, in the absence of other easily recognized distinctions, have always divided the physician from the quack; while the patentees themselves have forgotten, that they have invented neither the drug they administer, nor the machine by which they administer it, nor even the idea of its application to the purposes for which they use it. Surely the vapor deserves the name they give it.

Dr. Flagg of Boston has firmly resisted the attempt of the patentees to force this nostrum upon the dental profession, and we tender him our hearty thanks for his fearless and honest course. The pretensions set up by Dr. Begelow, that it is proper and right to patent inventions in mechanical dentistry, is too absurd, too offensive to the good sense, honorable feeling and correct practice of respectable dentists to be worthy of refutation. There is no conventional understanding among us to permit such patent-mongering. We think indeed that we could name quite as many respectable physicians who are disgracing their profession by vending nostrums, as he can discover dentists of acknowledged standing, engaged in such practices.

We trust that our readers will be cautious how they credit the statements of cases sent forth by interested parties, preparatory to receiving satisfactory evidences of the virtues of ethereal inhalation. Experiments here have been by no means satisfactory, Prof. Smith of the Maryland University permitted the administration of the vapor to three patients, it not only did not succeed, but acted injuriously, and we have simi lar information from other quarters.

The last number of the Boston Medical and Surgical Journal contains a legal opinion from a lawyer upon the subject of the patent in this case. The writer shows that things *patented* cannot remain *secret;* moreover, that inventions in medicine and surgery are not excluded from this kind of protection. Nobody has disputed either of his propositions. The question is as to the right of patenting the article in question, and about this he says nothing—probably because he has no defence to offer.

The effects resulting from, or at least, liable to result from the inhalation of the vapor of sulphuric ether, are, in our opinion, more to be dreaded than the pain of almost any surgical operation. We would, therefore, caution our professional brethren against the use of an article capable of producing such sudden, powerful and dangerous effects.

